# Abstracts/Sommaires/Zusammenfassungen

**DOI:** 10.1155/S1463924681000485

**Published:** 1981


### Abstracts Sommaires Zusammenfassungen

### Page 173

**An evaluation of the Kodak glucose/BUN analyzer including experience with proposed testing protocols**
H.M. Barbour *et al*

An evaluation of the analysis of serum urea and plasma glucose on the Kodak Ektachem GLU/BUN®analyzer is described. It incorporates the experimental design and statistical methods outlined in the Proposed Standard for Establishing Performance Claims (PSEP) (1) for clinical chemical methods PSEP-2, 3 and 4 produced by the National Committee for Clinical Laboratory Standards (NCCLS). These documents include details of baseline performance, precision and method comparison studies. Additionally, material from the Wellcome control scheme was analysed by both test and comparative methods. The results are presented and discussed together with comments gained from our experience in using the NCCLS documents. The results from the statistical analysis are employed to make some judgements concerning method acceptability.

**Evaluation de L'analyseur glucose/BUN de Kodak ainsi que l'xpérience avec les procés verbaux suggérés**

Une évaluation de l'analyse de l'urée de sérum et la glucose de plasma avec l'analyseur de Kodak EKTACHEM GLU/BUN® est décrite. Elle inclut une description des conditions expérimentales ainsi que des méthodes statistiques tel que cela a été décrit dans les standards proposés pour établir la performance (PSEP) pour méthodes cliniques PSEP-2, 3 et 4 produits par le comité national pour standards de laboratoires cliniques (NCCLS). Ces méthodes incluent les details en ce qui concerne la ligne de base, la précision et des études de comparaison de méthodes. En plus du matériel du plan de contrôle Wellcome a été analysé par la méthode étudiée ainsi que par des méthodes de comparaison. Les résultats sont présentés et discutés. Des commentaires sont faits à la suite des expériences faites utilisant les documents de la NCCLS. Les résultats de l'analyse statistique sont employés pour juger si la méthode est acceptable.

**Eine Beurteilung des Kodak Glukose/BUN Analysators und Erfahrungen mit dem vorgeschlagenem Test-Protokoll**

Eine Beurteilung der Analyse Harnstoffserum und Plasma-Glukose mit dem Kodak EXTACHEM GLU/BUN® Analysator wird beschrieben. Sie umfasst die experimentelle Gestaltung und statistischen Methoden die in "Proposed Standard for Establishing Performance Claims (PSEP)" für klinisch-chemische Methoden PSEP-2, 3 und 4 des National Committee for Clinical Laboratory Standards (NCCLS) erläutert werden. Diese Dokumente umfassen Details zu Basislinienverhalten, Präzision und Methodenvergleichstudien. Zusätzlich wurde Material des Wellcome Kontrollplans mit Test- und Vergleichs-Methoden analysiert. Die Resultate werden zusammen mit Kommentaren, die wir aus unserer Erfahrung in der Benützung der NCCLS Dokumente herleiten, präsentiert und diskutiert. Die Resultate der statistischen Analyse werden für einige Beurteilungen im Bezug auf die Methodenakzeptabilität verwendet.

### Page 178

**Method comparisons, influence of the number, distribution and range of samples on performance claims**
D. Burnett *et al*

The protocol produced by the National Committee for Clinical Laboratory Standards (NCCLS) for establishing performance claims for clinical chemistry methods, PSEP-4(1) provides guidelines for method comparison studies. Sample distributions are suggested for a number of analyties which should total a minimum of one hundred duplicate analyses by the test and comparative methods. This paper uses data obtained in comparison of two glucose methodologies and studies the effect of number, distribution and range of samples on the estimates from linear regression analysis which are used in calculating the tolerance limits and estimates of total error which it is suggested should provide a basis of manufacturers performance claims [1].

Analysis of the glucose data indicates that the one hundred samples recommended by the NCCLS provides a good assessment of linear regression parameters, fifty data points could provide adequate information if the range and distribution are carefully chosen.

**Comparaison de méthodes, influence du nombre, de la distribution et de la plage des échantillons sur les performances prétendues**

Le protocol produit par le comité national pour standards en laboratoires cliniques (NCLS) pour établir des assertions de performance, PSER-4(1) indique les lignes générales pour études de comparaison de méthodes. La distribution d'échantillons est suggérée pour un nombre de substances et un minimum de cent analyses en double avec la méthode sous étude et la méthode de comparaison est recommandé. Cette publication utilise les données obtenues lors de la comparaison de deux méthodologies de glucose et étudie les effets du nombre, du domaine et de la plage des échantillons sur les estimations de l'analyse à régression linéaire qui sont utilisées pour calculer les limites de tolérance et pour estimer l'erreur totale qui devraient fournir une base pour les performances que le manufacturier prétend. [1].

L'analyse des données de glucose indique que le nombre de cent échantillons recommendé par le NCCLS permet un bon jugement des paramètres de la régression linéaire, cinquante points de données pourraient fournir une information adéquate à condition que la plage et la distribution soient choisis soigneusement.

**Methodenvergleich und Einfluss von Zahl, Verteilung und Bereich der Proben auf die Leistungsspezifikationen**

Das Protokoll PSEP-4, das vom National Committee for Clinical Laboratory Standards (NCCLS) für die Festlegung von Leistungsdaten für klinisch-chemische Methoden erstellt wurde, enthält Richtlinien für Methodenvergleichsstudien. Probenverteilungen werden vorgeschlagen für eine Anzahl von Analysenproben, welche mindestens 100 betragen soll und für die Doppel analysen mit Test- und Vergleichs-Methoden durchgeführt werden. Die vorliegende Arbeit benutzt Daten die beim Vergleich von zwei Glukosemethodologien erhalten wurden und untersucht die Wirkung der Zahl, Verteilung und Bereich der Proben auf die Resultate der linearen Regressionsanalyse, welche für die Berechnung der Toleranzgrenzen und der Abschätzungen des Gesamtfehlers, welche als Basis für die vom Hersteller angegebenen Leistungsdaten vorgeschlagen sind [1].

Die Analyse der Glukosedaten deutet darauf hin, das die 100 Proben, die durch das NCCLS empfohlen werden, für die Beurteilung der linearen Regressionsparameters eine gute Beurteilungsgrundlage abgeben, währenddem 50 Datenpunkte dann genügend Information enthalten, wenn Bereich und Verteilung sorgfältig ausgewählt werden.

### Page 182

**Microcomputer control and data system for automated multiple flow injection analysis**
J.F. Brown *et al*

A control and data system has been developed for automated multiple flow injection analysis (AMFIA) using a Rockwell

**Contrôle par microordinateur et système de données pour un système automatique à multiple "flow injection"**

Un système de contrôle et de données a été développé pour l'analyse automatique à multiple "flow injection" (AMFIA) en

**Steuerungs- und Auswertungs-System mit Mikrocomputer für die automatisierte, multiple kontinuierliche Injektions-Analyse (FIA)**

Ein Steuerungs- und Daten-System für die automatisierte, multiple kontinuierliche Injektions-Analyse (AMFIA) wurde ent-

AIM 65 microcomputer. The system provides "turnkey" operation of the standard, titration and dilution configurations of AMFIA hardware, acquires and stores peak area and width data for up to 120 samples and provides hard copy of data and calculations. The "turnkey" program can be altered with editing utilities that are part of the operating system, or the operating system can be modified by use of the AIM 65 programming languages. It is simple to make extensive alterations to the program. Very little training is required to perform routine assays in the "turnkey" mode: in a research environment, two levels of programming are available to modify or expand AMFIA operation.

utilisant un microordinateur Rockwell AIM 65. Le système permet opération à "tour de clé" des configurations standard AMFIA (titrage et dilution), reçoit et garde en mémoire les surfaces et largeurs de pic de 120 échantillons et imprime des compte-rendus des données et des calculs. Le progamme "tour de clé" peut être changé à l'aide d'un programme éditeur qui fait partie du système, ou alors à l'aide de langues de programmation du AIM 65. Des changements importants sont faciles à effectuer. L'entraînement nécessaire pour le travail de routine dans le mode, "tour de clé" est minime. Pour la recherche deux niveaux de programmation sont à disposition pour changer ou expandre les possibilités du système AMFIA.

wickelt, dass mit einem Rockwell AIM 65 Mikrocomputer arbeitet. Das System offeriert "schlüsselfertigen" Betrieb der Standard-, Titrations- und Verdünnungs-Konfigurationen der AMFIA Instrumentierung, erfasst und speichert Kurvenflächen und Linienbreiten für über 120 Proben und liefert einen Ausdruck der Daten und Berechnungen. Das "schlüsselfertige" Programm kann mit einem Editierprogramm, das Bestandteil des Betriebssystems ist, modifiziert werden, oder das Betriebssystem kann mit Hilfe der AIM 65 Programmiersprachen modifiziert werden. Die Durchführung extensiver Aenderungen des Programms ist sehr einfach. Sehr wenig Uebung ist nötig um Routineanalysen in der "schlüsselfertigen" Betriebsart durchzuführen, für Forschungsaufgaben sind zwei Stufen von Programmierung verfügbar um Aenderungen oder Ergänzungen des AMFIA Betriebs durchführen zu können.

### Page 187

**Control programming without language: automation of vitamin $B_6$ analysis**
J.F. Brown *et al*

A system has been developed for automated multi-sample HPLC determinations of vitamin $B_6$. Novel features of this implementation are the microcomputer interface to system elements and the use of self-programming software. The microcomputer operates sample and column-control valves, an "intelligent" detector and a programmable integrator/calculator. Teaching the system new protocols involves manually operating the equipment throughout one analysis with the microcomputer in "learn" mode; subsequent samples may then be analysed automatically. The system illustrates a general method of automating many laboratory instruments.

**Programmation de contrôle sans langage: automation de l'analyse automatique de la vitamine $B_6$**

Un système a été développé pour l'analyse automatique multi-échantillon par HPLC de la vitamine $B_6$. La nouveauté de ce système est l'interface par microordinateur des différents éléments et l'utilisation d'un software auto-programmable. Le microordinateur opère les vannes d'échantillons et de contrôle de colonne, un détecteur "intelligent" et un intégrateur/calculateur programmable. Pour apprendre au système une nouvelle méthode, le système doit être opéré manuellement pendant une analyse avec le microordinateur dans le mode "learn = apprendre": Les échantillons suivants peuvent ensuite être analysés automatiquement. Le système illustre une méthode générale d'automation pour beaucoup d'instruments de laboratoire.

**Steuerprogramme ohne Benützung einer Programmiersprache: Automation der Vitamin $B_6$ Analyse**

Es wird ein System vorgestellt, dass für die automatisierte HPLC Bestimmung von Vitamin $B_6$ in vielen Proben entwickelt wurde. Neuartige Eigenschaften dieser Realisierung sind das Mikrocomputer-Interface zu den Systemelementen und die Benützung von sich selbstprogrammierender Software. Der Mikrocomputer betätigt Proben-und Kolonnen-Steuerventile, einen "intelligenten" Detektor und einen programmierbaren Integrator/Rechner. Um dem System neue Protokolle beizubringen muss lediglich während einer Analyse das System manuell betätigt werden, wobei der Mikrocomputer auf "lernen" gesetzt ist; folgende Proben können daraufhin automatisch analysiert werden. Das System illustriert eine allgemeine Methode für die Automatisierung vieler Laborinstrumente.

### Page 191

**Continuous monitoring using thiocyanate ion-selective electrodes**
Takashi Korenaga

On the continuous monitoring of thiocyanate in environmental water samples, feasibilities of thiocyanate ion-selective electrodes with liquid and solid membranes are presented by investigating the practical problems. With regard to industrial waste waters containing about 2000 p.p.m. chloride, only a liquid membrane electrode could be applied to the continuous monitoring of thiocyanate, and the results obtained with the automatic method using a liquid membrane electrode agreed with those with the manual spectrophotometric method using ferric nitrate with a few positive difference.

**Contrôle continu utilisant une electrode spécifique pour le thiocyanate**

La possibilité du contrôle continu du thiocyanate dans l'eau avec des électrodes sélectives à membranes liquides et solides est étudié sur des échantillons pratiques. En ce qui concerne des eaux industrielles polluées contenant plus de 2000 ppm de chlorure, uniquement une électrode à membrane liquide a pu être utilisée pour le contrôle continu du thiocyanate. Les résultats comparaient bien avec la méthode manuelle spectrophotométrique qui utilise le nitrate ferreux.

**Kontinuierliche Ueberwachung mit Thiocyanate ionen-selektiven Elektroden**

Für das Gebiet der kontinuierlichen Ueberwachung von Thiocyanat in Umwelt-Wasserproben werden Einsatzmöglichkeiten für Thiocyanationenselektiven Elektroden mit flüssigen und Festkörper-Membranen dargestellt anhand der Untersuchung der praktischen Probleme. In Bezug auf industrielles Abwasser, das ungefähr 2000 p.p.m. Chlorid enthält, konnte nur eine Flüssigmembran-Elektrode für die kontinuierliche Ueberwachung von Thiocyanat verfügbar gemacht werden. Die Resultate, die mit der automatisierten Methode auf der Basis einer Flüssigmembran-Elektrode erhalten werden, stimmen bis auf eine kleine positive Abweichung mit denjenigen der manuellen spektrophotometrischen Methode auf der Basis Eisennitrat überein.

### Page 196

**Present sample identification systems**
H. Keller

The paper discusses the importance and problems associated with the accurate identification of samples in clinical laboratories. Essential features for any effective identification system are outlined and four generalised systems (two indirect systems and two direct systems) are described. Specific labelling and identification schemes such as barcoding and the SILAB-system are also discussed.

Abstracts continued on p. 210

**Systèmes actuels pour l'identification d'échantillons**

Cet article discute l'importance et les problèmes associés à l'identification précise d'échantillons dans les laboratoires cliniques. Les propriétés essentielles de tout systeme d'identification efficient sont détaillées et quatre systèmes (deux indirects et deux directs) sont décrits. Des schémas spécifiques de codage et d'identification, tels que le système SILAB et le codage par traits sont discutés.

**Heutige Probenidentifikations-Systeme**

Die Arbeit bespricht die Bedeutung und Probleme, die mit einer zuverlässigen Identifikation der Proben klinischen Laboratorien zusammenhangen. Wichtige Eigenschaften für ein effizientes Identifikations-System werden auseinandergesetzt, und vier verallgemeinerte Systeme (zwei indirekte und zwei direkte Systeme) werden beschrieben. Spezifische Etikettierung und Identifikationsschemata wie z.B. Strichcode und das SILAB-System werden ebenfalls besprochen.

